# Influence of liquid and gas flow rates on sulfuric acid mist removal from air by packed bed tower

**DOI:** 10.1186/1735-2746-9-20

**Published:** 2012-12-10

**Authors:** Mohammad Javad Jafari, Roohollah Ghasemi, Yadollah Mehrabi, Ahmad Reza Yazdanbakhsh, Majid Hajibabaei

**Affiliations:** 1 Department of Occupational Health Engineering, Shahid Beheshti University of Medical Sciences, Tehran, Iran; 2 Department of Epidemiology, Shahid Beheshti University of Medical Sciences, Tehran, Iran; 3 Department of Environmental Health Engineering, Shahid Beheshti University of Medical Sciences, Tehran, Iran

**Keywords:** Packed tower, Scrubber, Sulfuric acid, Flow rate

## Abstract

The possible emission of sulfuric acid mists from a laboratory scale, counter-current packed bed tower operated with a caustic scrubbing solution was studied. Acid mists were applied through a local exhaust hood. The emissions from the packed bed tower were monitored in three different categories of gas flow rate as well as three liquid flow rates, while other influencing parameters were kept almost constant. Air sampling and sulfuric acid measurement were carried out iso-kinetically using USEPA method 8. The acid mists were measured by the barium-thorin titration method. According to the results when the gas flow rate increased from 10 L/s to 30 L/s, the average removal efficiency increased significantly (p < 0.001) from 76.8 ± 1.8% to 85.7 ± 1.2%. Analysis of covariance method followed by Tukey post-hoc test of 92 tests did not show a significant change in removal efficiency between liquid flow rates of 1.5, 2.5 and 3.5 L/min (p = 0.811). On the other hand, with fixed pressure loss across the tower, by increasing the liquid/gas (L/G) mass ratio, the average removal efficiency decreased significantly (p = 0.001) from 89.9% at L/G of <2 to 83.1% at L/G of 2–3 and further to 80.2% at L/G of >3, respectively. L/G of 2–3 was recommended for designing purposes of a packed tower for sulfuric acid mists and vapors removal from contaminated air stream.

## Introduction

A large number of epidemiologic studies have shown that elevated levels of several air pollutants, including acid aerosols and sulfates are correlated with an increased prevalence of pulmonary disease. Strong inorganic acid mists containing sulfuric acid (H_2_SO_4_) have been reported to correlate with lung and laryngeal cancer in humans [1-3] and is recognized as a human carcinogen by US National Toxicology Program [4]).

Sulfuric acid is a strong acid widely used in different applications. Low volatility, high reactivity, high acidity, high corrosivity, and high affinity for water are its’ specific chemical characteristics [3,5]. In the atmosphere and inventing stacks, it is formed from sulfur dioxide, sulfur trioxide and oleum (a combination of sulfur trioxide and sulfuric acid used in industry) [6]. Sulfuric acid mists and vapors may also be emitted into the atmosphere directly from its numerous industrial applications.

The control of sulfuric acid mist and vapor are much concerned from environmental and occupational health points of view. On the other hand, conducting experimental tests using such a strong acid is a challenging work. According to USEPA, packed bed wet scrubbers or packed towers can be referred as acid gas scrubber when it is used to control inorganic gases [7,8], but it is not clearly considered for removal of sulfuric acid. The operation of a packed wet scrubber is based on absorption.

Absorption is the process of transfer of a gaseous pollutant from a gas phase to a liquid phase [9]. In air pollution control, absorption involves the removal of objectionable toxic gases from the process stream and dissolving them in a liquid. The absorption process can be categorized as physical and chemical absorption. Physical absorption occurs when the absorbed compound dissolves in liquid. If the absorbed compound reacts with the liquid or reagents chemical absorption occurs [10].

Removal efficiencies vary for each pollutant-solvent system and with the type of gas absorber used. While the most absorbers have removal efficiencies in higher than 90%, the packed tower absorbers may achieve efficiencies as high as 99.9% for some pollutant-solvent systems [8,11]. Since the sizes of acid mist and vapor differ from the molecules of gases, different behaviors are expected when sulfuric acid mist and vapor are introduced into a packed tower. The investigations of Thomas showed that the absorption performance of a packed tower decreases with increase of H_2_SO_4_ in liquid content [12]. Therefore it is important to apply caustic solution to achieve a higher absorption performance in a packed tower when removing sulfuric acid mist and vapor from air. The performance of such a packed tower is not clear.

The type of gas and liquid flow through an absorber may be counter-current, cross-current, or co-current. Counter-current flow is the most commonly installed design. The waste gas stream enters at the bottom of a counter-current flow absorber column and exits at the top, while the solvent stream enters at the top and exits at the bottom. This leads the counter-current designs to provide the highest theoretical removal efficiency because liquid with the lowest pollutant concentration contacts gas with the lowest pollutant concentration. This maximizes the average driving force throughout the column leading to the highest absorption. In addition, counter-current design is more suitable when the air pollutant loading is higher and usually requires lower liquid to gas ratios than co-current designs [11].

Packed towers which are columns filled with packing materials provide a large surface area to facilitate contact between the liquid and gas. Achieving high removal efficiencies, handling high liquid rates, and consuming relatively lower water requirements than other types of gas absorbers are the main advantages of packed towers. However, high system pressure drops, high clogging and fouling potential, and extensive maintenance costs, as well as higher installation operation and wastewater disposal costs of packed bed absorbers may be considered as their disadvantages. Solvent costs, pump and fan power requirements and operating costs associated with replacing damaged packing should also be considered for packed towers [11].

Many factors (including toxic pollutant solubility in the absorbing liquid, liquid to gas ratio (L/G), pressure drop, collection efficiency), and construction details of the absorber (such as packing plates, liquid distributors, entrainment separators and corrosion-resistant materials) are involved in the design of a packed tower. More details are discussed by [13-15].

The objective of the present research was to conduct a series of bench-scale testing of a single stage packed bed scrubber employing sodium hydroxide and water scrubbing solutions to study the influences of different gas and liquid flow rates( Q_gas_ and Q_liq_) on sulfuric acid mist removal by a packed tower. The role of liquid and gas flow rates is discussed in present paper.

## Materials and methods

### Packed tower scrubbers

**Figure 1:**
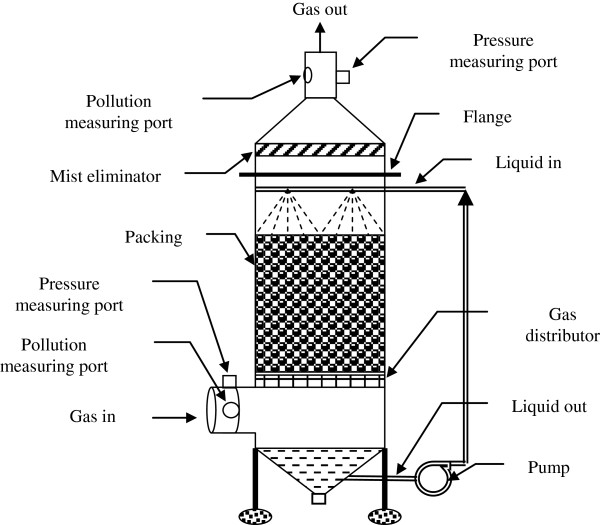
Counter-current single stage packed tower scrubber.

A counter-current single stage packed tower scrubber at bench scale was constructed from black iron painted with anti-corrosion paint (Figure 1). The inner diameter of the scrubber was 20cm with packing depth of 60cm. The scrubbing bed was randomly packed using ceramic intalox saddle packing with 2.5cm diameter. Packed tower scrubber was comprised of a column shell, liquid distributor, packing material and packing support.

All basic components as explained by [11], except mist eliminator were used in the constructed packed tower. According to Theodore, a demister is only used when the gas velocity in the tower is higher than 18 m/s (60 ft/s) [15]. Since the gas velocity was far less than that, the mist eliminator was not installed in the packed tower. Mist eliminators were placed in the gas outlet to prevent any liquid droplet carry-over from bed to the outlet stack. Ceramic intallox saddles used in the present study provided a large contact surface for scrubber solution and the contaminated air stream [16].

### Acid mist preparation

Strong acid mist preparation, such as sulfuric acid, through direct injection of acid into the inlet air in order to get sub-micron mists is a challenging task. Vaporizing the acid through heating is a safer alternative method that compiles with the USEPA definition of sulfuric acid mist. According to USEPA the sulfuric acid mist includes not only liquid mists but also sulfur trioxide (SO3) and sulfuric acid vapor [11].

In present study, spraying of diluted acid (5-10%) in a mixing box was not practically successful to get a relatively high concentration of acid mist in the air. Heating of high concentrated sulfuric acid in a laboratory erlen was only able to produce a concentration of less than 5 mg/m^3^ acid mist in the air. The third attempt using an erlen with a small fan in it as well as the fourth attempt using autoclove to vaporize diluted acid also failed to get concentrated acid mist in the air.

In the final attempt, high concentration acid was vaporized in beshers under a hood using an electric heater. The vapor was exhausted by a hood to the packed tower. Acid mist and vapor of as high as 1200 mg/m^3^ (in the air) was obtained. It was possible to use up to 6 beshers each on a 6 sectional heater.

### Air sampling and acid mist measurement

The concentration of sulfuric acid mist was measured using USEPA method 8 at inlet and outlet of the scrubber through pollution measuring ports (Figure 1). For this purpose, gas samples were extracted isokinetically from the inlet and outlet ducts connected to the packed tower scrubbers. The sulfuric acid mist including SO3 and SO2 were separated, and both fractions were measured by the barium-thorin titration method. The barium ions react preferentially with sulfuric ions in the solution to form a highly insoluble barium sulfate precipitate. When the barium has reacted with all sulfate ions, it’s excess reacts with the thorin indicator to form a metal salt of the indicator to give color change [17].

A sampling train similar to those recommended by USEPA method 8 was constructed. The train was similar to the method 5 train, except that it was not heated and the filter position was different.

Construction details described in APTD-0581 were considered for sampling train [18]. SKC sampling pumps and standard laboratory equipments were used. Four Greenburg-Smith design impingers, as recommended by method 8 of USEPA, were used for air sampling.

Metering system, a barometer and gas density determination equipment were all the same as those in method 8, sections 2.1.8, 2.1.9, and 2.1.10, respectively. All sampling equipments were the same as those recommended by method 8 of USEPA.

Four isoporpanol samples, obtained from different commercial sources were tested according to method 8, in order to select the appropriate peroxides free one.

### Air flow and pressure loss measurements

Air flow required for tests were produced by a variable flow rate fan model HVDLT-MK2, manufactured by UK air flow Co. A low pressure loss venturi with an accuracy of 95-99% was used to measure the flow rate. Calibrating tests were performed to choose the most precise air flow measuring device. A pitote tube with an accuracy of 98% was used to measure the pressure drop at packed tower in each test. Air flow rates were also double checked using pitote tube along with an inclined manometer.

### Liquid flow and pH measurements

The scrubbing liquid was re-circulated through a pump (Figure 1), and was set to desired rates using a valve. Liquid pH was regulated using sodium peroxide. The liquid flow rate and pH were measured 5 times during each sampling period, using a flow meter and a pH meter, respectively. The pH meter was calibrated prior to each sampling test.

### Other parameters measurement

Other parameters including, air and water temperature as well as barometric pressure, were also measured during each sampling period as described in method 8 of USEPA.

## Results

### Efficiency vs. gas flow rate

The overall results (mean ± standard error) of 92 tests conducted at different gas flow rates while, other parameters including packing material, bed height, scrubbing liquid pH, input acid mist concentration and liquid flow rate, were kept almost constant, are shown in Table 1.

**Table 1:** **Sulfuric acid mist removal efficiency**$\bar{X}\pm \mathrm{SE}$**vs. gas flow rate**

**Q**_**gas**_**(L/s)**	**Test No.**	**t**_**residence**_**(s)**	**Q**_**liq**_**(L/min)**	**L/G**	**pH**_**liq**_	**H**_**packing**_**(cm)**	**ΔP (pa)**	**C**_**in**_**(mg/m**^**3**^**)**	**Efficieny(%) Mean ± SE**
10	23	1.92 ± 0	2.6 ± 0.2	5.9 ± 0.4	9.0 ± 0.8	61	75.1 ± 22.8	236.0 ± 35.9	76.8 ± 1.8
20	40	0.96 ± 0	2.6 ± 0.1	2.9 ± 0.1	7.8 ± 0.6	61	167.9 ± 4.3	233.6 ± 27.5	83.6 ± 1.2
30	29	0.64 ± 0	2.6 ± 0.1	1.9 ± 0.1	8.3 ± 0.5	61	204.0 ± 9.0	242.9 ± 37.3	85.7 ± 1.2

The results from 23 air samples collected at the gas flow rate of 10 L/s showed that the minimum, average and maximum efficiencies at a gas flow rate of 10 L/s are 67.0%, 76.8 ± 1.85% and 99.0 %, respectively. The results from 40 tests showed that when the flow rate increased to 20 L/s, the above measures increased to 68.6, 83.6 ± 1.23% and 99.3%, respectively. With gas flow rate increasing to 30 Lit/s, the results from 29 tests showed that minimum, average and maximum efficiencies changed to 67.6, 85.7% ± 1.23% and 98.9%.

**Figure 2:**
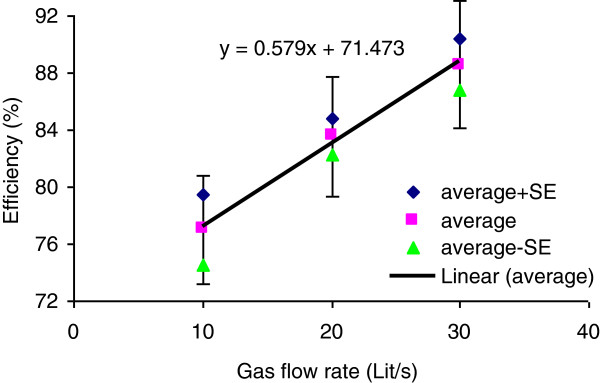
Sulfuric acid mist removal efficiency vs. gas flow rate at fixed pressure loss (Err Bar: 5%).

According to Table 1, as the gas flow rate increased, the pressure loss across the tower bed increased by 171.6% which may disturb the direct influence of gas flow rate on removal efficiency. Therefore, in order to remove the effect of pressure loss on the efficiency, analysis of covariance (ANCOVA) method was employed followed by Tukey post-hoc test. The results revealed significant increase in removal efficiency between gas flow rate of 10 and 20 L/s (p = 0.003). However, no significant change was observed between 20 and 30 L/s. Figure 2 depicts the average ± standard errors vs. gas flow rate while thepressure loss across the tower bed was kept fixed.

### Efficiency vs. scrubbing liquid flow rate

The results from 92 tests conducted at different scrubbing liquid flow rates while other parameters including gas flow rate, scrubbing liquid pH, bed height, packing material, input acid mist concentration and pressure loss were kept almost constant, showed that when the scrubbing liquid flow rate increased from 1.5 L/min to 2.5 L/min, the average removal efficiency decreased, but when the liquid flow rate further increased from 2.5 to 3.5 L/min, the average efficiency increased (Table 2). Higher concentration of sulfuric acid mists applied with 2.5 L/min scrubbing solution may have led to higher removal efficiency.

**Table 2:** **Sulfuric acid mist removal efficiency**$\bar{x}\pm \mathrm{SE}$**vs. scrubbing liquid flow rate**

**Q**_**liq**_**(L/min)**	**Test No.**	**t**_**residence**_**(s)**	**Q**_**gas**_**(Lit/s)**	**L/G**	**pH**_**liq**_	**H**_**packing**_**(cm)**	**ΔP (pa)**	**C**_**in**_**(mg/m**^**3**^**)**	**Efficieny(%) Mean ± SE**
1.5	24	1.1 ± 0.1	20.4 ± 1.6	1.9 ± 0.2	9.5 ± 0.5	61	142.8 ± 11.1	226.9 ± 32.8	83.6 ± 1.8
2.5	36	1.05 ± 0.1	20.8 ± 1.1	3.0 ± 0.2	7.5 ± 0.6	61	157.9 ± 8.3	258.6 ± 32.6	81.0 ± 1.5
3.5	32	1.12 ± 0.1	20.6 ± 1.4	4.7 ± 0.4	8.3 ± 0.6	61	164.0 ± 13.0	217.0 ± 29.9	83.5 ± 1.2

The results from 24 tests conducted with liquid flow rate of 1.5 L/min showed that the minimum, average and maximum efficiencies at a liquid flow rate of 1.5 L/min were 67.0, 83.6% ± 1.85% and 98.9%, respectively. The results from 36 tests showed that when the liquid flow rate increased to 2.5 L/min, the above measures changed to 67.6%, 81.1 ± 1.54% and 99.3%, respectively. With further increase of the liquid flow rate to 3.5 L/min, the results from 32 tests showed that minimum, average and maximum efficiencies changed to 70.2%, 83.5 ± 1.2% and 95.8%, respectively.

**Figure 3:**
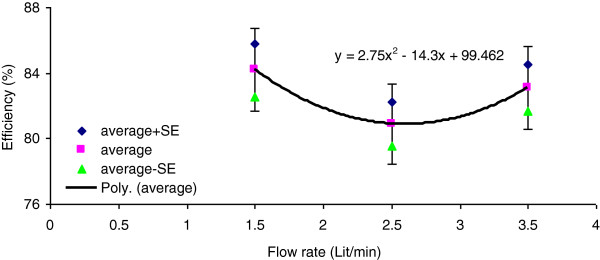
Removal efficiency vs scrubbing liquid flow rate at fixed input concentration (Err Bar: 3%).

According to Table 2, as the scrubbing liquid flow rate increased the input concentration of sulfuric acid mist and vapor changed by 19.2% which may disturb the direct influences of scrubbing liquid flow rate on removal efficiency. Therefore, in order to remove the influences of input concentration on the efficiency, analysis of covariance (ANCOVA) method was employed followed by Tukey post-hoc test. The results did not show a significant change in removal efficiency between liquid flow rates of 1.5, 2.5 and 3.5 L/min (p = 0.811). This result was expected for such a chemical absorption of sulfuric acid mists in caustic scrubbing liquid. Figure 3 depicts the average ± standard errors vs. gas flow rate while the input concentration of acid mists applied to the tower is kept fixed at 247 mg/m^3^.

### Efficiency vs. liquid to gas ratio

The results from 92 tests conducted at different gas flow rates, input acid mist concentrations, scrubbing liquid flow rates and pH, while other parameters including bed height and packing material were kept fixed, showed that as the scrubbing liquid to gas mass ratio increased, the removal efficiency decreased (Table 3).

**Table 3:** **Sulfuric acid mist removal efficiency**$\bar{x}\pm \mathrm{SE}$**vs. liquid to gas flow rate**

**L/G**	**Test No**	**t**_**residence**_**(S)**	**Q**_**gas**_**(Lit/s)**	**Ql**_**iq**_**(Lit/min)**	**pH**_**Liq**_	**H**_**packing**_**(cm)**	**ΔP (pa)**	**C**_**in**_**(mg/m**^**3**^**)**	**Efficien(%) Mean ± SE**
<2	27	0.75 ± 0.03	26.7 ± 0.9	1.9.9 ± 0.1	8.9 ± 0.5	61	178.7 ± 5.8	240.8 ± 40.7	85.6 ± 1.3
2-3	31	0.88 ± 0.05	23.2 ± 1	2.8 ± 0.1	6.9 ± 0.7	61	184.8 ± 10.5	259.5 ± 31.1	84.0 ± 1.5
>3	34	1.58 ± 0.08	13.5 ± 0.8	2.9 ± 0.1	9.0 ± 0.6	61	111.9 ± 9.3	213.7 ± 27.7	78.8 ± 1.4

The results from 27 tests conducted with liquid to gas mass flow ratio of <2 showed that the minimum, maximum and average removal efficiencies were 67.6%, 85.6 ± 1.31% and 98.9%, respectively. The results from 31 tests conducted with a liquid to gas mass flow ratio of 2–3 revealed that these efficiencies were 68.6%, 84.1 ± 1.55% and 99.3%, respectively. The results from 34 tests with liquid to gas mass flow ratio of >3 showed that the minimum, average and maximum efficiencies were 67.0%, 78.8 ± 1.40% and 99.0%, respectively (Table 3).

According to Table 2, as the scrubbing L/G mass flow ratio increases, the gas flow rate decreased and the scrubbing liquid flow increased as well. By increasing the liquid flow, the pressure loss increases slightly while by decreasing the gas flow rate, the pressure loss across the tower decreases. The overall influences of these two parameters leads to an increase in pressure loss followed by its decrease. The results showed that as it was expected, lower removal efficiency has been obtained at lower pressure losses across the tower. Tukey post-hoc test showed that the significantly different (p = 0.001) pressure loss across the tower between L/G ratio of <2 and >3 as well as 2–3 and >3 led to significantly different (p = 0.025) removal efficiencies. The same test revealed that a non-significantly different pressure loss (p = 0.89) between L/G of <2 and 2–3 led to a non- significantly different removal efficiency (p = 0.739).

**Figure 4:**
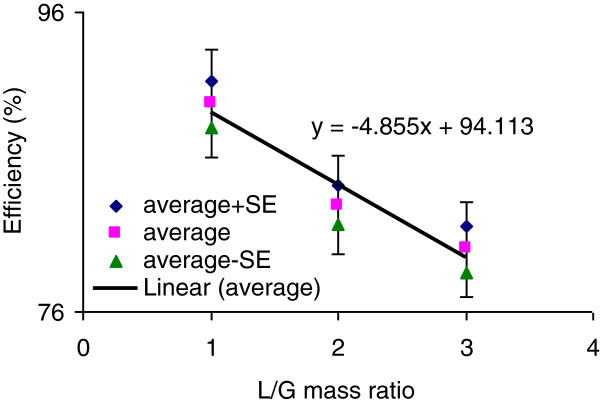
Removal efficiency vs. liquid to gas mass ratio at fixed pressure loss (Err Bar: 4%).

The statistical analysis of 92 tests in which pressure loss in the packed tower was kept fixed showed that by increasing L/G mass ratio, the average removal efficiency decreased (Figure 4) significantly (p = 0.001); more details are described below.

The results of 27 tests conducted with L/G of <2 (with pressure loss kept fixed) showed that the average removal efficiency was 89.9 ± 1.55% (Figure 4). The results from 31 tests conducted with L/G between <2 and >3, showed that the average removal efficiency was 83.1 ± 1.31%. According to the results of 34 tests conducted with L/G of >3, the average removal efficiency was 80.2 ± 1.53% (Figure 4).

## Discussion

### Sulfuric acid mist removal efficiency

Although, when the high removal efficiencies of acid gases are required, the wet scrubbers are often the technology of choice, but SO2 which finally changes to SO3 and H2SO4, in its removing process is a more difficult pollutant to be wet scrubbed. Traditionally, wet scrubber designs call for 90–95% removal efficiency for SO2 [14]. Since aerosols are not treated in a wet scrubber, thus lower removal efficiencies are expected for sulfuric acid mists through scrubbing. The particle sizes produced in this study by boiling sulfuric acid liquid is expected to be in the range of 20–1000 μm in diameter [13]. With such large aerosol sizes, lower removal efficiencies are inevitable.

The average sulfuric acid mist removal obtained in present study ranged from 76.8%-85.7%. According to Ceilcote air pollution control Co, the removal efficiency of their packed towers for sulfuric acid mists have been in the range of 85%-90% for a packed tower with packing depth of 91.4 cm and 98-99% for a packed tower with packing depth of 152.4cm. Jiuan reported efficiencies of 98%-99% for sulfuric acid mist in a packing depth of 122cm and >99% for a packing depth of 183 cm [8].

When high removal efficiency is expected, the mist eliminator plays a significant role as well. Since the present packed tower was not equipped with mist eliminator, thus lower removal efficiencies were expected [16]. Forming of large size aerosols, lack of demister, relatively low pressure loss and lower packing depth are expected to be the main reasons for lower removal efficiency in present study.

### Efficiency vs. gas flow rate

In some instances, elevated pressures are used to give additional driving force of the pollutant into the liquid stream to accelerate the mass transfer from gas phase to the liquid phase. This may be achieved through increasing the input flow rate, application of smaller packing material or increasing packed bed height. The results from the present study showed that even with low air velocities tested here, the removal efficiency increased meaningfully.

The ANCOVA statistical test showed that there was not a significant difference between average efficiencies at 20 and 30 L/s gas flow rates (p = 0.502), but there was a significant difference between average efficiencies at 10 and 20 L/s (p = 0.003), as well as between 10 and 30 L/s (p = 0.001) gas flow rates.

Table 1 shows that as the gas flow rate increased from 10 to 30 L/s, the average removal efficiency increased by 8.9%. Higher gas flow rates led to higher turbulent flow, introducing higher energy to the gas which consequently leads to higher removal efficiencies [7]. Low gas flow rate of 10 L/s leads to an interfacial velocity of 0.318 m/s which is a laminar flow. The gas molecules are not able to penetrate into the liquid with such a low dynamic energy. The interfacial velocities tested in present study were 0.318, 0.636 and 0.955 m/s, respectively at different flow rates tested. These velocities are much lower than those recommended by ACGIH (e.g. 1 to 1.5 m/s) for packed towers [7]. Higher removal efficiencies are expected with even higher gas flow rates tested in present study.

Statistical analysis of covariance with fixed pressure drop across the tower bed also revealed that even with fixed pressure drop across the bed, the average removal efficiency increases by 8.6% as the gas flow rate increases from 10 to 30 L/s, which is mainly due to the increased driving force at higher air velocities.

### Efficiency vs. liquid flow rate

According to the texts, mass transfer from gas into liquid is dependent on the physical properties of the gas liquid matrix (e.g., diffusivity, viscosity, density) as well as the conditions of the scrubber system (e.g., temperature, pressure, gas and liquid mass flow rates). Absorption of a pollutant is enhanced by lower temperatures, greater liquid gas contact surfaces, higher liquid gas ratios, and higher concentration of the pollutant in the gas phase (or, alternately, lower concentration of the pollutant in the liquid phase). The results from the present study showed that when the input concentration of the pollutant decreased, a significant reduction in mass transfer from gas into liquid was observed.

According to Table 2, when the liquid flow rate increased from 1.5 L/min to 2.5 L/min, the removal efficiency decreased for 2.5%. Further increase in liquid flow rate increased the removal efficiency back by 2.5%. Tukey post hoc statistical analysis showed that the variation of the average efficiency due to the variation of liquid flow rate is not significant. This is probably because at 2.5 L/min of liquid flow, the input concentration has been increased from 226.9 to 258.6 mg/m^3^. Higher input concentration has unbalanced the molar equilibrium of acid moles and scrubbing liquid.

Therefore, in order to remove the influences of input concentration on the efficiency, analysis of covariance (ANCOVA) method was employed followed by Tukey post-hoc test. The results did not show a significant change in removal efficiency between liquid flow rates of 1.5, 2.5 and 3.5 L/min (p = 0.811). This result was expected for such a chemical absorption.

The ANCOVA method followed by Tukey post-hoc test with fixed input acid mist concentration revealed that when the liquid flow rate increased from 1.5 to 2.5 L/min, the average removal efficiency decreased by 3.3%. Further increase in liquid flow rate from 2.5 to 3.5 L/min increased the average removal efficiency by 2.2%. The variation of removal efficiency vs. liquid flow rate at fixed input concentration was not significant. The variation of input concentration during this part of the study was not favorable. Tests with fixed input concentrations are suggested.

### Liquid to gas ratio

Absorption of a pollutant is enhanced by higher liquid gas ratios and higher concentrations of the pollutant in the gas phase (or, alternately, lower concentration of the pollutant in the liquid phase). The results from conducted tests in present study showed that the sulfuric acid mist removal efficiency is higher at liquid to gas mass ratios of less than 2. According to the results, by increasing this ratio, the efficiency decreases.

The Tukey post hoc statistical analysis showed that there is not a significant difference between average removal efficiency with L/G mass ratio of 2-3and >3, (p = 0.025), while there is a significant difference between average removal efficiency with L/G mass ratio of <2 and 2–3 (p = 0.739). Statistical analysis also showed that there is a significant difference between average removal efficiency with L/G mass ratio of <2 and >3 (p = 0.025).

The results from the present study do not agree with those presented in texts in which by increasing L/G, the efficiency is expected to increase. L/G ratios were obtained from different tests conducted with different air and liquid flow rates as well as different input pollutant concentrations, which could be the main reason for such a contrary result. This part of the experiment should be conducted with only liquid flow rate changing, while other influencing parameters are kept constant.

L/G mass flow ratio for packed towers, reported by some institutes such as ACGIH is usually in the range of 0.6 to 1.2 [7]. Higher L/Gs applied in the present study could be another reason for its contrary results.

The liquid to gas ratio is a key parameter to start designing of a packed tower [15]. According to the results of present study, a liquid to gas mass flow ratio in the range of 2 to 3 is suggested for design purposes of a packed tower to remove sulfuric acid mists from contaminated air streams.

## Competing interests

The authors declare that they have no competing interests.

## Authors’ contributions

MJJ was the Head of the research team, the main contributor to the project, supervised the whole project and wrote the paper. RG was the main assistant and conducted the experimental works. YM supervised the statistical analysis. ARY assisted in experimental works. MHB was the second assistant and contributed in laboratory works. All authors read and approved the final manuscript.
